# Meningococcal arthritis and myopericarditis: a case report

**DOI:** 10.1186/s12879-017-2845-3

**Published:** 2017-12-06

**Authors:** Lloyd Steele, Katie Bechman, Eoghan De Barra, Charles Mackworth-Young

**Affiliations:** Charing Cross Hospital, Imperial College Healthcare NHS Trust, Fulham Palace Road, London, W6 8RF UK

**Keywords:** Neisseria meningitidis, Arthritis, Infectious, Meningococcal infections, Pericarditis

## Abstract

**Background:**

We report the first adult case of *Neisseria meningitidis W-135* presenting with meningococcal arthritis and myopericarditis concomitantly, without other classical features of meningococcal disease.

**Case presentation:**

A 67-year-old Caucasian man presented with acute-onset polyarthralgia, myalgia, and fever. On examination he had polyarticular synovitis. An electrocardiogram (ECG) demonstrated ST-elevation in leads I, II, III, aVF, and V2-V6 without reciprocal depression, and a high-sensitivity troponin level was significantly elevated. Cardiac magnetic resonance (CMR) imaging on day five of admission demonstrated patchy pericardial enhancement. *Neisseria meningitidis W-135* was isolated from both synovial fluid and blood cultures. The clinical outcome was favourable with intravenous ceftriaxone and myopericarditis treatment (colchicine and ibuprofen).

**Conclusions:**

We conclude that this is a rare case of disseminated *Neisseria meningitidis W-135* presenting with acute polyarticular septic arthritis and myopericarditis, without other classical features of systemic meningococcal disease. The earlier described entity of primary meningococcal arthritis (PMA) can present in patients with meningococcal bacteraemia, and may not be distinct from disseminated meningococcal disease, but rather an atypical presentation of this.

**Electronic supplementary material:**

The online version of this article (10.1186/s12879-017-2845-3) contains supplementary material, which is available to authorized users.

## Background

Focal infections with meningococcus such as septic arthritis and pericarditis are often poorly recognised as presentations of meningococcal disease. We report the first adult case of *Neisseria meningitidis W-135* presenting with meningococcal arthritis and myopericarditis concomitantly, without other classical features of meningococcal disease.

## Case presentation

A 67-year-old Caucasian man presented with a one-day history of acute-onset polyarthralgia, myalgia, and fever. Apart from one episode of diarrhoea there were no other symptoms. He denied any precipitating trigger including recent sexual contact. He was born in South Africa but had lived in the UK for seven years prior to his presentation to our department. He had not travelled outside of the UK in that time. He was a non-smoker and worked as a glass collector in a kitchen. There was no history of intravenous drug use. His past medical history was significant for Ménière’s disease.

His vital signs on admission showed a tachycardia (heart rate 101 beats per minute) and fever (temperature 38.0 °C), but were otherwise normal (respiratory rate 18breaths/min, SpO2 97%, and blood pressure 107/79 mmHg). On examination he had polyarticular synovitis involving both knees (with moderate effusions), the right shoulder, left wrist, and right third proximal interphalangeal joint. There were no skin rashes and no peripheral lymphadenopathy. Cardiovascular examination was unremarkable.

Laboratory investigations revealed lymphopaenia (0.2 × 10^9^ cells/L), thrombocytopaenia (128 × 10^9^ cells/L), anaemia (haemoglobin 105 g/L), an acute kidney injury (creatinine 129 μmol (89% increase from baseline)), and an acute phase response (C-reactive protein (CRP) 309 mg/L and erythrocyte sedimentation rate (ESR) 39 mm/h). An autoimmune screen (rheumatoid factor, antinuclear antibody, and anti-neutrophil cytoplasmic antibody) was negative. Complement 3, complement 4, CH50 level, and alternate pathway complement function were normal. Urine microscopy revealed only scanty red cells and <50 white cells per cubic millimetre.

**Fig. 1:**
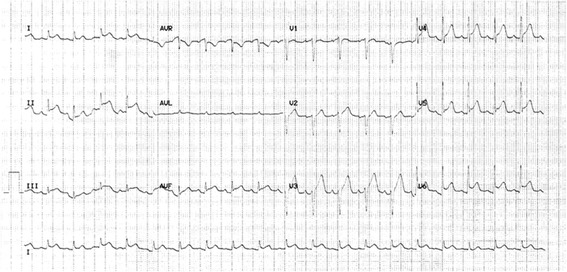
Twelve lead electrocardiogram showing ST-elevation in leads I, II, III, aVF, and V2-V6 without reciprocal depression

**Fig. 2:**
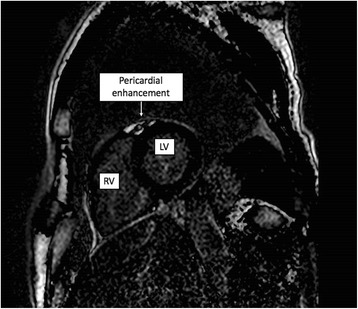
Cardiac magnetic resonance (CMR) short-axis stack image showing the left (LV) and right ventricles (RV), with pericardial enhancement

An electrocardiogram (ECG) demonstrated ST-elevation in leads I, II, III, aVF, and V2-V6 without reciprocal depression (Fig. 1). A high-sensitivity troponin I level was significantly elevated at 33,169 ng/L (0-34 ng/L) with a peak of 48,140 ng/L at 12 h post-admission. An urgent echocardiogram using a pocket-sized ultrasound device (V-scan) on day one demonstrated mild-moderate left ventricular impairment with no regional wall motion abnormalities. Cardiac magnetic resonance (CMR) imaging on day five of admission demonstrated patchy pericardial enhancement with no obvious myocardial enhancement (Fig. 2).

Aspiration from the right knee yielded a purulent fluid, and microscopy revealed Gram negative cocci, monocytes, and polymorphs. *Neisseria meningitidis W-135* was isolated from both synovial fluid and blood cultures. The serotype was confirmed at Public Health England’s Meningococcal Reference Unit using standard methods [1] (see Additional file 1).

The patient was commenced on treatment for myopericarditis with colchicine and ibuprofen after Cardiology assessment. Intravenous ceftriaxone 2 g twice per day was commenced after review by the Infectious Diseases team, initially due to a suspected diagnosis of disseminated gonococcal infection. Azithromycin treatment was not given due to reports of causing colchicine toxicity. The patient did not develop nuchal rigidity or other signs of meningism or meningococcaemia. His fever resolved by day three with a subsequent improvement in the inflammatory response. Troponin levels were monitored daily and fell sharply, normalising after 5 days of therapy, and a departmental echocardiogram at this point demonstrated improved ventricular function. After two weeks of treatment the lymphopaenia and thrombocytopaenia had resolved, and renal function had returned to baseline. Intravenous ceftriaxone was then switched to oral ciprofloxacin 500 mg twice per day which was continued for a further week.

His musculoskeletal symptoms were slower to improve despite regular non-steroidal anti-inflammatory medication and physiotherapy. Synovitis at the shoulder and wrist persisted for approximately 6 weeks. He developed symptoms of carpal tunnel syndrome on day 6, which had not resolved by outpatient review at 8 weeks. At clinic review he stated his intention to return to South Africa on a permanent basis. He was advised to consider carpal tunnel release in South Africa if his symptoms continued.

## Discussion

Acquisition of meningococcus is usually through very close contact with respiratory secretions or saliva [2, 3]. It can asymptomatically colonise the nasopharynx, as in 8–25% of healthy individuals [2, 4], or cause invasive disease [2]. The mechanisms that lead from colonisation to invasive disease are still incompletely understood, but are thought to be a result of environmental conditions, host susceptibility, and meningococcal virulence factors [2, 5]. The degree of virulence of *N. meningitidis* is influenced by capsule expression, which itself is used to define the individual serogroups [2]. Although 13 distinct serogroups exist, the majority of invasive meningococcal diseases are caused by only six of these: A, B, C, W, X, and Y [2, 3, 5]. In Europe, serogroups B and C cause the majority of invasive disease cases [2]. Since 2009, the number of serogroup W cases in England has been increase year-on-year, from 19 in 2008/2009 to 176 in 2014/2015 (a quarter of all laboratory-confirmed meningococcal cases that year), with a rise in the hypervirulent ST-11 stain [6]. Interestingly, during the twenty-first century, serogroup W has become the predominant cause of invasive disease in all age groups in South Africa, in place of serogroup A [7, 8]. However, the overall incidence of meningococcal disease in South Africa is at an all-time low, with an average incidence in the population of 1 case per 100,000 population [9].

The two most common clinical presentations of invasive meningococcal disease are meningitis and the often fatal syndrome of acute meningococcaemia [5]. The ability of *N. meningitidis* to cause disease beyond these presentations is often overlooked [10]. Other presentations include pericarditis, conjunctivitis, panophthalmitis, pneumonia, urogenital tract infections, and arthritis [2].

Arthritic involvement is not an uncommon feature of invasive meningococcal disease, occurring in up to 11% of adult cases [11]. The majority of this occurs in patients with acute meningococcaemia with direct haematogenous seeding of circulating bacteria [11, 12]. Two other clinical patterns of meningococcal arthritis are also described [11, 12]: an immune-mediated arthritis, characterised by sterile purulent effusions, and an acute septic arthritis in patients without meningitis or the classical syndrome of meningococcaemia, defined as the combination fever, rash, and hemodynamic instability [11]. Earlier studies have defined this as primary meningococcal arthritis (PMA) [11, 12]. However the classification of PMA is somewhat confusing. The synovium is positive for meningococcus in 90% of cases [12], but *N. meningitidis* is also cultured from the blood in 40% of cases [13]. Additionally the infection is likely to originate outside of the joint, and thus characterising cases with bacteraemia as PMA may be problematic and a general description of meningococcal septic arthritis may be more appropriate.

PMA remains a rare diagnosis, with only 46 cases reported up to 2016 [14]. Presentation of PMA may be very similar to that of other forms of septic arthritis [12]. It is most commonly monoarticular [15], with the knee the most commonly involved joint [12, 14, 16], but can also be oligoarticular or polyarticular [12, 16]. Causative serotypes are group B, 30–38%; group C, 33–36%; and group W-135, 13–29% [15, 17]. The majority of cases due to group W-135 are in children [15, 18–21], with only one case reported in an adult [13]. Our patient did not have meningism or the features of acute meningococcaemia, and thus his condition fits with a diagnosis of PMA.

In addition to joint involvement, our patient presented with myopericarditis. Meningococcal pericarditis is an unusual but well-described complication of meningococcus, occurring in 3–19% of cases [22–24]. As with articular involvement, it can be classified into three categories: a process secondary to disseminated disease (purulent; culture-positive; associated with widespread, clinically-manifested meningococcal disease); a reactive immune-mediated process (immunological, late-onset, culture-negative); or a primary infection (purulent, culture-positive, but without evidence of meningeal involvement or clinical manifestations of meningococcaemia) [25]. Common features include ECG abnormalities (93%), chest pain (84%), and fever (69%) [22]. Cardiac tamponade is also a recognised complication, and in such cases pericardiocentesis, a pericardial window, or even a pericaridiotomy may be required [25]. Features which can suggest concomitant myocardial involvement (myocarditis) include dyspnoea, palpitations, fatigue, and a troponin rise [26], with the latter being a prominent part of our patient’s presentation.

The prognosis for arthritic involvement in meningococcal arthritis is excellent. In one study although 33% of patients were treated with steroids for up to several months due to persisting symptoms, all patients recovered and there was no joint deformity or impairment at long-term follow-up [27]. The literature suggests that recovery from acute meningococcal myocarditis can occur within 3–6 days [28, 29], a similar time course to that observed in our case.

Preventing invasive meningococcal disease has been an issue of great importance in public health. An effective conjugate group C vaccine was introduced in the UK in 1999, and then conjugate group A vaccines in the African meningitis belt beginning in 2010 [30], but throughout this time developing a vaccine against serogroup B had been a challenge. However, recently, there have been reports of early efficacy for the serogroup B vaccine in the UK [31], which now has a comprehensive programme against serogroups A, B, C, W, and Y. Ongoing surveillance will be needed to assess the longer-term impact of this.

## Conclusions

We report a rare case of disseminated *Neisseria meningitidis W-135* presenting with acute polyarticular septic arthritis and myopericarditis, without other classical features of systemic meningococcal disease. The earlier described entity of PMA can present in patients with meningococcal bacteraemia, and may not be distinct from disseminated meningococcal disease, but rather an atypical presentation of this. Meningococcus should be included in the differential diagnosis of focal infections including septic arthritis and myopericarditis in the absence of meningitis or meningococcaemia.

## Additional file

Additional file 1: Public Health England Meningococcal Reference Unit User Manual. (PDF 816 kb)

## Abbreviations

CMR: Cardiac Magnetic Resonance
CRP: C-Reactive Protein
ECG: Electrocardiogram
ESR: Erythrocyte Sedimentation Rate
PMA: Primary Meningococcal Arthritis
