# Supplementary material for: Meningococcal arthritis and myopericarditis: a case report
Source: BMC Infect Dis. 2017 Dec 6;17:751. doi: 10.1186/s12879-017-2845-3 (PMC5718011; doi:10.1186/s12879-017-2845-3)
Supplement: Additional file 1: — Public Health England Meningococcal Reference Unit User Manual. (PDF 816 kb) [file 12879_2017_2845_MOESM1_ESM.pdf]

Public Health England

Meningococcal Reference Unit

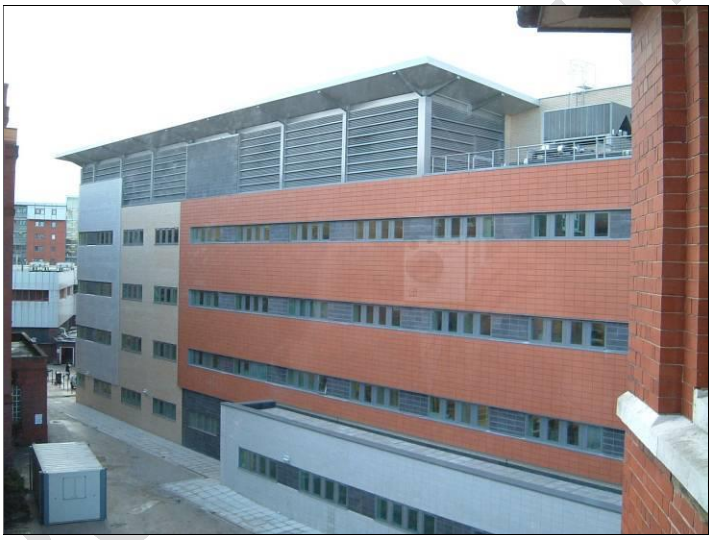

User Manual

September 2015

Contains Information on References Services for:

- *Neisseria meningitidis* isolate characterisation
- Polysaccharide antigen detection
- *Neisseria meningitidis* (Meningococcal) DNA detection by PCR (*Streptococcus pneumoniae* detection by PCR)
- Vaccine response – (pre- and post- immunisation)

**DX address**:	DX 6962410<br>Manchester 90 M
**Courier address (Real postcode)**	Meningococcal Reference Unit<br>Public Health England<br>Manchester Public Health Laboratory<br>Manchester Medical Microbiology Partnership<br>Clinical Sciences Building 2<br>Manchester Royal Infirmary,<br>Oxford Road<br>Manchester, UK **M13 9WL**
**Postal address (virtual postcode)**	Meningococcal Reference Unit<br>Public Health England<br>Manchester Medical Microbiology Partnership<br>**PO Box 209**<br>Clinical Sciences Building 2<br>Manchester Royal Infirmary,<br>Oxford Road<br>Manchester, UK **M13 9WZ**
Telephone	#44(0)161 276 6757
Fax	#44(0)161 276 5744
Out-of-hours Telephone	#44(0)161 276 1234<br>and ask for Medical Microbiologist on-call

Controlled Document

Authorised By:

Dr E B Kaczmarski (Head of Meningococcal Reference Unit)

Effective Date: September 2015

CONTENTS

MENINGOCOCCAL REFERENCE UNIT INTRODUCTION 4

ENHANCED SURVEILLANCE, FROM SEPTEMBER 2015 5

MRU CONTACT DETAILS 6

SUMMARY OF SERVICES AND RESOURCES 7

SERVICES AVAILABLE 8

URGENT CULTURE SPECIMENS 13

MRU PCR INVESTIGATIONS PERFORMED 17

PCR TURNAROUND TIMES - AVAILABILITY OF PCR RESULTS 18

URGENT PCR SPECIMENS 18

KEY FACTORS AFFECTING SPECIMEN PERFORMANCE 22

TURNAROUND TIMES AND PRICE LIST 23

SPECIMEN AND SAMPLE SUBMISSION GUIDELINES 24

LABELLING YOUR SPECIMENS MATTERS 24

RELEVANT CLINICAL INFORMATION 24

COMPLAINTS 26

FAXING AND EMAILING REPORTS CONTAINING PATIENTS' DATA 26

COMPLIANCE WITH THE HUMAN TISSUE ACT – SUBMITTING SAMPLES FROM DECEASED PEOPLE 27

MRU RECOGNITION OF CALDICOTT RECOMMENDATIONS 27

KEY REFERENCES 28

CHARITIES AND PUBLIC INFORMATION CONTACT DETAILS 30

Controlled Document

Meningococcal Reference Unit Introduction

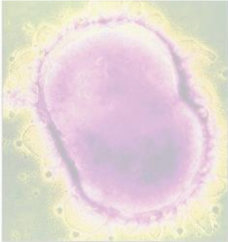

The PHE Meningococcal Reference Unit (MRU) for England and Wales has been situated in Manchester since 1978. Originally established to provide phenotypic characterisation of meningococci isolated from cases of invasive meningococcal disease (IMD) in laboratories throughout the country, the nature and scope of this confirmation and surveillance activity has widened as has the range of tests available.

The MRU re-located from Withington Hospital, Manchester to Central Manchester Foundation Trust (CMFT) in March 2003 as an integral part of the Manchester Medical Microbiology Partnership (MMMP).

The MRU is part of the PHE Reference Microbiology Services Division and works closely with other parts of the PHE particularly the Immunisation, Hepatitis and Blood Safety Department to optimise meningococcal disease ascertainment through enhanced surveillance often supported by PHE Health Protection teams.

The MRU has been a world leader in developing and making nationally available tests for non-culture case confirmation of meningococcal infection by PCR. Initially designed to identify the major disease causing serogroups (A, B, C, Y and W), the test repertoire has been extended to provide more detailed additional characterisation utilising molecular techniques including DNA sequencing of isolate genes (directly from clinical specimens where possible),and since 2010, whole genome sequencing of case isolates.

Optimised surveillance, along with serological studies performed in the PHE Vaccine Evaluation Unit co-located within the MMMP at MRI were key elements in supporting and monitoring the successful introduction of meningococcal serogroup C conjugate vaccine in the UK, the introduction of serogroup A conjugate vaccine in sub-Saharan Africa and have contributed significantly to establishing the international reputation of the MRU.

In addition to providing confirmatory laboratory services, staff from the MRU can advise on investigation and management of individual cases and outbreaks.

The MRU and PHE Colindale have been active in the establishment of a network of national and regional reference laboratories which are collaborating to harmonise and optimise surveillance throughout Europe and sharing this experience with other interested groups in the Americas and Oceania. This has resulted in the establishment of the European Meningococcal Disease Society (EMGM). The MRU supports work-packages in the European Centre for Disease Control (ECDC) Invasive Bacterial Diseases Laboratory Network (ECDC IBDLabNet): with particular regard to the provision of External Quality Assessment (EQA) in collaboration with PHE Colindale and UK NEQAS colleagues. The MRU was until recently a global referee laboratory for the World Health Organisation (WHO) Invasive Bacterial Diseases EQA and since 2013 has (with PHE and UK NEQAS colleagues) managed WHO EQA panels for WHO Regional and Sentinel Laboratories.

Controlled Document

Enhanced surveillance, from September 2015

MenB national childhood immunisation programme

From 1 September 2015, the MenB (Bexsero®) vaccination will be added to the NHS Childhood Immunisation Programme in England to help protect children against this devastating disease that which can cause meningitis and septicaemia (blood poisoning), which are serious and potentially fatal illnesses. Babies will be offered the MenB vaccine with the other routine vaccinations at two months, four months and 12-13 months of age. Vaccinating babies at these times helps protect them when they are most at risk of developing MenB disease. Infants under 1 year of age are most at risk of MenB and the number of cases peak at around 5 or 6 months of age.

https://www.gov.uk/government/collections/meningococcal-b-menb-vaccination-programme

National enhanced surveillance of vaccination programmes targeting invasive meningococcal disease in England,
PHE publications gateway number: 2015294. Version 1.1 Date 01/09/2015.
https://www.gov.uk/government/uploads/system/uploads/attachment_data/file/457723/MeningoEnhancedSurveillancePlan_01092015_v1.1.pdf

Meningococcal disease: guidance, data and analysis.
The characteristics, diagnosis, management, surveillance and epidemiology of meningococcal disease.
https://www.gov.uk/government/collections/meningococcal-disease-guidance-data-and-analysis

The national surveillance protocol for invasive meningococcal disease (IMD) in England has been extended in recognition of:

1. Changes to the meningococcal group C (MCC) conjugate vaccination programme, including the removal of the infant MCC dose at 4 months and the introduction of an adolescent MCC dose in June 2013.
2. The emergency introduction of a quadrivalent conjugate vaccine against meningococcal groups A, C, W, and Y (MenACWY) for 14-18 year-olds in August 2015 in response to a national outbreak of a hypervirulent MenW.

There has been a slow and steady increase in invasive meningococcal disease due to capsular group W (MenW) since 2009. This increase appears to be due to expansion of a single hyper-virulent strain belonging to clonal complex 11 (cc11) and has been observed across all regions. MenW cases were not associated with travel, indicating that this strain is now endemic in England. Since 2011, MenW cases have been diagnosed across all age groups and are associated with higher case fatality than the more common meningococcal group B (MenB) cases.

https://www.gov.uk/government/publications/meningococcal-disease-laboratory-confirmed-cases-in-england-and-wales

Controlled Document

MRU Contact Details

General MRU Result enquiries	
Identification, phenotypic characterisation (serogroup, serotyping, subtyping), molecular characterisation (*porA* sequencing) and susceptibility testing of isolates<br>Antigen detection<br>PCR<br>Requests for molecular epidemiology<br><br>Database management	**Initial contact for most MRU enquiries:**<br>**Tel 0161 276 8788 or 8854 or 6757**<br><br>**Dr Stephen J Gray PhD.,**<br>**Lead BMS3**<br>Tel: #44(0)161 276 6764<br>steve.gray@phe.gov.uk<br><br>**Mr Anthony Carr, BMS2**<br>tony.carr@phe.gov.uk<br><br>**Dr Lynne Newbold PhD., BMS2**<br>lynne.newbold@phe.gov.uk
**Medical Enquiries**	
Patient investigation and clinical advice<br>Interpretation of results<br>Outbreak investigation and management advice	**Dr Edward Kaczmarski FRCPath**<br>**Head of MRU**<br>Tel: #44(0)161 276 5699<br>Mobile: 07774243886<br>ed.kaczmarski@phe.gov.uk
**Other Key Staff**	
Vaccine evaluation, research and development<br>Vaccine response assessment<br>Proposed research projects	**Professor Ray Borrow PhD., FRCPath,**<br>**Deputy Unit Head of MRU**<br>**Head of PHE Vaccine Evaluation Unit (VEU)**<br>Tel: #44(0)161 276 8850<br>ray.borrow@phe.gov.uk
PCR diagnosis of *N. meningitidis*<br>Service and molecular research projects	**Dr Malcolm Guiver PhD., FRCPath,**<br>**Head of Molecular Diagnostics**<br>Tel: #44(0)161 276 8833<br>malcolm.guiver@phe.gov.uk<br><br>**Mrs Laura Grice, BMS2**<br>laura.grice@phe.gov.uk
Other sources of information<br><br>PHE Immunisation	**Dr Mary Ramsay Consultant Epidemiologist**<br>Immunisation, Hepatitis and Blood Safety Department.<br>Public Health England<br>61 Colindale Avenue,<br>London, NW9 5EQ<br>Tel: #44(0)20 8200 6868<br>mary.ramsay@phe.gov.uk<br><br>**Dr. Shamez Ladhani**<br>shamez.ladhani@phe.gov.uk<br>**Dr. Sema Mandal**<br>sema.mandal@phe.gov.uk

Controlled Document

Summary of Services and Resources

- Clinical advice for case and outbreak investigation and management
- Meningococcal isolate confirmation and characterisation
- Meningococcal DNA detection by PCR for non-culture case confirmation
- Molecular characterisation of meningococcal isolates and non-culture (DNA positive only) material
- Technical laboratory advice and support for large scale investigations
- Meningococcal vaccine evaluation
- Determination of response to meningococcal vaccination
- Collection of >50,000 phenotypically characterised meningococcal isolates
- Collection of >2000 whole genome sequenced meningococcal case isolates
- Selected collection of clinical samples (under HTA licence)
- Computerised database of laboratory confirmed cases
- Computerised epidemiological database of all submitted isolates since 1995
- Support for collaborative scientific projects and audits

Controlled Document

Services Available

How to obtain MRU services

The routine submission of samples for either PCR investigation or isolate confirmation with a completed PHE MRU Request Form following the specimen acceptance policy (below) will suffice in most instances.

<u>**Telephone contact**</u>

For general enquiries: 0161 276 8788 or 8854 or 6757
The MRU laboratory is available Monday – Friday, 09:00 to 17:00

The MMMP call centre utilises an automated filter system. **Use option 1 for authorised (completed) MRU results**.

Clinical enquiries will be directed to an available consultant.

<u>**Weekend enquiries**</u>

For urgent clinical enquiries, particularly those occurring out of hours, weekends or on bank holidays please contact Dr Ed Kaczmarski mobile phone or contact via the consultant medical microbiologist rota through Central Manchester Foundation Trust switchboard on 0161 276 1234.

Out of hours specimens

Clinical specimens for PCR investigation must be received at the MRU by 10.00am weekdays to be tested the same working day.

Arrangements to accept <u>**urgent couriered samples**</u> for PCR or other investigations <u>**must**</u> be agreed with the MRU **before** the samples are despatched. Failure to do so may result in the specimen(s) not being tested in a timely fashion.

If a delivery is expected to arrive after 5.30pm, Monday – Friday, at weekends, or on Bank Holidays, it should be left at the CMFT Autolab reception (ground floor of Clinical Sciences Building 2). Out of hours access to the Clinical Sciences Centre is granted to couriers via the security intercom. The entrance is situated at the North entrance of the Clinical Sciences Building 2.
(If entering the MRI site from Hathersage Road, it is on the left after passing under the link bridge).

Controlled Document

<u>Urgent couriered specimens for PCR investigation</u>* should be addressed to

> **"Virologist* On-Call" (*if out of hours*)**
>
> **Meningococcal Reference Unit – URGENT SPECIMEN**
> **Public Health England**
> **Manchester Medical Microbiology Partnership**
> **Clinical Sciences Building 2**
> **Manchester Royal Infirmary**
> **Oxford Road**
> **Manchester M13 9WL**

***If live cultures are being submitted for serogoup or other characterisation please state "Microbiologist" On-Call** (*if out of hours*)

<u>Transport containers and documentation</u>

It is the responsibility of senders to comply with the current transport legislation and safety recommendations.

Samples (including clinical samples for PCR and viable cultures) **must** only be submitted in packaging appropriate for the transport of biological substance category B (**UN3373**).

Refer to IATA Cat B packing instructions "**pi650**".
http://www.iata.org/whatwedo/cargo/dgr/documents/dgr52_pi650_en.pdf

All packages must be correctly labelled (addressed) and be accompanied by the appropriate documentation.
The specific MRU request form should be used and completed in accordance with the specimen acceptance policy – see specimen sample submission guidelines (below).

Controlled Document

Routine Investigations

***Neisseria meningitidis* isolate characterisation**

Submission of a viable culture of *N. meningitidis* or query Neisseria -

Please send cultures from **all** positive sites of IMD cases: sterile (CSF, blood, joint fluids etc.) and in addition any from non-sterile sites (nose, and throat)

Where possible pure, **viable cultures inoculated on agar slopes: chocolate (heated) blood agar, blood agar or Dorset egg slopes** after establishing growth by overnight incubation at 37°C.
On occasion, it may be necessary to submit an unincubated culture. This can save time but requires a heavy inoculum to ensure survival in transport. Please indicate on the request form if the material (slope) has not been incubated.
Short-term storage of sloped cultures is optimal at 30°C if there are delays before submission.

Agar slopes (in bijoux with screw-capped lid) are preferred for growth and safety but (short) **transport swabs in Amies** medium are accepted. Note: the presence of charcoal may interfere with direct, rapid agglutination reactions if urgent results are required.

The use of **liquid Transport swabs is not recommended** as there is a risk of leakage in transport and greater risk of aerosols upon opening at receipt. Liquid transport systems for (automated) clinical investigation may not be appropriately designed single organism culture shipment: If in doubt please discuss with MRU staff before submission.

It is acceptable to submit **frozen liquid suspensions of viable cultures** (in accordance with safety considerations and correct transport documentation) following prior discussions with MRU staff to ensure material is either sub-cultured upon receipt or appropriately stored. This is the preferred method for large studies.

Unusual samples
Silica gel packages (WHO Manual, 2nd Edition, 2011) can be accepted following specific prior arrangement with MRU staff.

Freeze-dried cultures can be accepted following specific prior arrangement with MRU staff

All preparations of potentially live samples for shipment should be in a microbiological safety cabinet.

Submission of non-viable cultures of *N. meningitidis*

Submission of viable organisms is always preferred but in some instances, cultures which are no longer viable may still be considered for characterisation by phenotypic and molecular based methods, after consultation with the MRU. A heavy inoculum of the inert material on a slope should be submitted with an appropriate request form stating

Controlled Document

that the culture is thought to be non-viable.

Species confirmation

Phenotypic confirmation of *Neisseria meningitidis* isolates based on morphology, conventional biochemical and serological reactions.

Epidemiological characterisation of strains

Phenotype:

(a) **Serogroup**: identification of capsular polysaccharide antigens by serological reactions: co-agglutination using *in-house* polyclonal antibodies, commercial slide agglutination, commercial latex antigen kits or preferably by an *in-house* dot-blot ELISA using monoclonal antibodies.

(b) **Serotype**: identification of porB (class 2/3) outer membrane proteins by a dot-blot ELISA using monoclonal antibodies.

(c) **Sero-subtype**: identification of porA (class 1) outer membrane proteins by a dot-blot ELISA using monoclonal antibodies.

Genotype:

(a) **Genogroup**: use of Taqman™ PCR assay where required enables identification of capsulated non-viable organisms: groups A, B, C, W or Y. PCR assays may also be used to identify the potential group of non-expressing (non-sterile site) isolates or to resolve occasional problematic serological characterisations.

(b) **Whole genome sequencing**: since July 2010 all meningococcal case isolates have been submitted for whole genome sequencing as part of IMD surveillance in England and are available in the Meningitis Research Foundation Meningococcus Genome Library (**http://www.meningitis.org/research/genome**) developed by Public Health England, the Wellcome Trust Sanger Institute and the University of Oxford as a collaboration. The project was funded by Meningitis Research Foundation from July 2010 to June 2013 and since July 2013 by PHE.

Molecular subtyping of isolates

Molecular characterisation of other potential typing targets and vaccine antigens such as ***fetA*** and ***fHbp*** (a component of Bexsero®) are now routinely identified through whole genome sequencing surveillance of all IMD case isolates.

Controlled Document

Antibiotic susceptibility testing

Minimum Inhibitory concentrations (MICs):

MICs are routinely determined and reported for all submitted isolates: penicillin, cefotaxime, rifampicin and ciprofloxacin using commercial gradient diffusion methodology.

Other antibiotic susceptibility tests (MICs) may be available upon request.

Please note that it is not unusual to determine a **"reduced susceptibility" to penicillin** for meningococcal case isolates; approximately 30% of case isolates show "reduced susceptibility" to penicillin as determined by current BSAC and EUCAST guidelines.

An unpublished review of the MRU MIC results for case isolates in the period epidemiological year 2014/15: using **BSAC** breakpoint interpretation v14.2015-01-01, 26/520 (**5%**) were **Resistant** (>0.25 mg/L), 140/520 (**27%**) were **Intermediate** (0.06 – 0.25 mg/L) and 354/520 (**68**%) were **Sensitive** (≤0.06 mg/L).
Following **EUCAST** guidelines v5.0 2015-01-01: 26/520 (**5%**) were **Resistant** (>0.25 mg/L) and 354/520 (**68%**) were **Sensitive** (≤0.06 mg/L).

Isolates with penicillin MICs > 0.5 mg/L should be submitted to the MRU.

In 2014/15, all case isolates of *N. meningitidis* were Sensitive to cefotaxime (and therefore ceftriaxone also).

Controlled Document

Turnaround times for isolate characterisation

Optimal turnaround times are conditional on receiving established pure cultures with appropriate documentation

Serogroup results for clinical isolates will be telephoned to the sending laboratory as soon as available – usually the following day after receipt following overnight sub-culture (Monday to Friday). The telephoned report is logged on the LIMS.

A '**final report'** comprising the serogroup, (serotyping and sero-subtyping) and antibiotic MIC profile are reported within 7 – 10 working days.

Contact the MRU for urgent weekend reporting or if rapid results are required e.g. for cluster or outbreak investigation.

The "additional report" of porA sequencing (VR1 and VR2) for case isolates has been discontinued since January 2014 and replaced by batched surveillance using whole genome sequencing..

Urgent culture specimens

In circumstances where urgent characterisation of an isolate is required, a provisional serogroup result can be available within two hours of receipt of an established culture.

When additional information of epidemiological importance such as serotyping and sero-subtyping is needed rapidly, a provisional phenotypic result can be available later the same working day if isolates are received before 10.00am, Monday to Friday.

Arrangements to process urgent isolate specimens should be made by telephone request to the MRU. This is particularly important if samples are likely to arrive at the MRU later than 17:00 Monday – Friday.

If required, molecular subtyping *(porA* and *fHBp* sequencing) requires a minimum of 3 working days from receipt.

Controlled Document

Meningococcal DNA detection by PCR

The MRU uses specific real-time PCR (ABI Taqman™) assays to confirm *N. meningitidis* (meningococcal disease) and determine the infecting capsular group where possible.

The MRU **meningococcal (*ctrA*) screening assay** is performed as part of a **four-component multiplex assay** also comprising: the meningococcal **serogroup B (*siaD*$_B$)** confirmatory assay, ***ply* (pneumolysin) for *Streptococcus pneumoniae* screen.**

The ***ctrA* PCR** assay confirms the presence of *N. meningitidis* DNA, (specifically for capsulated meningococci as *ctrA* is involved in the transfer of polysaccharide to the cell surface). [The addition of another reverse *ctrA* primer to the originally published assay (Corless *et al*., 2001) since 2003 has allowed for the additional detection of a small subset of meningococci not confirmed by the initial assay (Guiver *et al*., 2011; Gray *et al*., 2012)].

Specific serogroup confirmatory ***siaD* PCR** assays are based on the sialylation of the polysaccharide capsule for serogroups: **B, C, Y and W**. **Serogroup A** polysaccharide is chemically distinct and not sialylated hence requires the specific ***mynA*** assay.

An **internal control** is added before sample nucleic acid extraction and is a full process control. Results of assays are only reported if the control amplification is successful. The internal control is used to indicate inhibitory samples when compared to the assay run positive controls, (McHugh *et al*., 2015).

The pneumococcal screening PCR, *ply positive* (samples) cases are confirmed by use of the ***S. pneumoniae*** specific **autolysin (*lytA*) PCR**.

Charges

The PHE MRU meningococcal PCR assays are reference services for England (and Wales) epidemiology, and when performed are free of charge.

PCR requests from Northern Ireland are charged.

The pneumococcal assay component is currently free of charge as it was introduced to support the enhanced surveillance of pneumococcal disease post pneumococcal conjugate vaccine introduction.

What specimens to send for Meningococcal PCR

MRU meningococcal PCR assays have been validated for: **EDTA (whole blood)**, **CSF**, **serum**, **plasma** and **joint fluids**.

EDTA whole blood and CSF are the preferred specimens.

Plasma or serum can be examined however sensitivity may be compromised.

If coagulated bloods are submitted it is only possible to test the serum fraction.

If multiple samples are submitted by the submitting laboratory **EACH** should have a unique identifier (sender's reference number).

Controlled Document

EDTA blood (2.5 – 5 mL) sample collected on admission (see collection and timing for PCR specimens below) should be sent routinely to the MRU in the event that PCR confirmation is required. Smaller volumes (0.5 – 1mL) from infants and babies can also be examined.

Heparinised or citrated samples can be tested, but EDTA is preferred

CSF samples, if available, should be sent in addition to an EDTA blood sample. Definitive laboratory confirmation of meningococcal meningitis can only be made by analysis of a CSF sample.

Note that meningococcal and pneumococcal **meningitis** may only be accurately confirmed by testing a **CSF sample**. Negative EDTA blood PCR results cannot exclude meningitis.

Other specimens from normally sterile sites may be examined after prior consultation with the MRU and a blood and/or CSF specimen should accompany them if available.

The nucleic acid extraction processes are designed for fluid samples so there will be limited experience of unusual sample types. Positive results may be determined for such samples on the understanding that these should be considered “unvalidated”. Negative results for “unvalidated” samples should be treated with caution..

Enhanced surveillance of pneumococcal disease has included the successful screening of **empyema fluid** and other respiratory samples by the pneumolysin (*ply*) PCR and confirmation with the autolysin (*lytA*) PCR.

If **tissue samples** (or blocks) require examination they should only be submitted following discussion with MRU staff as they will require bespoke manual processing with concomitant increases in turnaround time.

Controlled Document

<u>Minimum volumes for PCR testing (DNA extraction)</u>:

Blood or fluids - the routine use of automated nucleic acid extraction systems requires a minimum 400 µL of blood. A larger volume is preferred in case repeat testing is required.

If smaller samples are submitted the fluid volume must be at least 100 µL. Small volumes will require dilution and separate extraction and this will increase turnaround times and may affect sensitivity.

CSF - 400 µL or more is preferred but small samples (50 µL) can be tested. The small volumes may require dilution prior to specific extraction and this will increase turnaround times and may affect sensitivity.
Low volume CSFs must be submitted in an appropriate sized container or tube. Whole CSF (ie. an uncentrifuged specimen) should be sent in small sterile containers such as a sterile 2mL screw capped vial rather than in universal containers.

Original CSF (uncentrifuged) or re-suspended CSF deposits are preferrable to CSF supernatants in order to increase sensitivity of detection.

If multiple CSF samples are submitted by the sender each should have a unique

identifier.

Collection and timing of samples for PCR testing:

The likelihood of a positive PCR result decreases as the interval of sampling after starting antibiotics lengthens. Blood samples for PCR taken more than 48 hours after commencement of antibiotic therapy are unlikely to give useful results.

It is recommended that samples for meningococcal PCR be submitted if collected less than 48 hours after onset, admission to hospital or administration of antibiotics.

The “48 hour” sampling advice is based upon over 15 years of PCR experience, the testing of 10,000s of samples in addition to studies of clinical samples and bacterial load, Hackett *et al*., (2002)**.**

CSF may remain “positive” for longer periods (Ragunathan *et al*., 2000)

Any specimens for PCR tests should be stored at 4°C and **not** frozen prior to transport. Freeze-thawing may reduce the likelihood of positivity with low genome copy samples and can result in cracked or broken containers.

Controlled Document

Other PCR investigations performed

Pneumococcal PCR assays

The samples found positive with the pneumolysin PCR (*ply*) screen in the four component multiplex assay are referred for confirmation using a pneumococcal specific autolysin (*lytA*) PCR. Samples found to be positive with both *ply* and *lytA* are reported as *S. pneumoniae* or pneumococcal PCR positive.

Samples that are only *ply* positive alone may indicate a streptococcal species. They are reported as pneumococcal PCR Negative.

Positive pneumococcal reports from blood in children under 2 years of age

It should be noted that there are reports in the literature of pneumococcal DNA detection (PCR positive) in the blood samples of children under the age of 2 years who are clinically well and this is thought to reflect asymptomatic carriage of *S. pneumoniae*. It is for that reason that all positive pneumococcal reports from bloods of children less than two years of age recommend clinical interpretation of the molecular results, (Dagan *et al*., 1998)

Hib PCRs

An additional and separate PCR assay to detect *Haemophilus influenzae* type b is available on request.

Other molecular detection assays

Situated within the MMMP molecular diagnostics department the MRU is able to request a variety of additional PCR-based assays including viral causes of meningitis (eg., Herpes simplex, enterovirus). The additional assays are not part of the free meningococcal service but may be added to requests at the time of submission or later. Should only limited amounts of unrepeatable samples be available this may be a cost-effective option. Nucleic acid extracts containing both DNA and RNA are available for rapid testing.
Additional viral PCRs will be charged.

Controlled Document

PCR turnaround times - availability of PCR results

PCR Negative and meningococcal group B positive results, are typically available within 24 hours of receipt.

Samples requiring meningococcal group (other than group B) and *S. pneumonia*e (pneumococcal PCR) confirmation are available within 48 hours.

Urgent samples may be processed more rapidly provided the laboratory is notified in advance of receipt.

All meningococcal PCR positive results will be telephoned following capsular group confirmation up to 17:30pm or as soon as possible on the morning of the next working day when printed reports will also be sent out.
Pneumococcal positives are reported similarly following species (*lytA*) confirmation.

It is useful to telephone the MRU (MMMP Call Centre) where a result is of particular urgency.

NB: Although hardcopy of the PCR results are sent to the Public Health England Centre (PHEC) it remains the responsibility of the requesting laboratory to inform their local CCDC or Health Protection Team (HPT) of positive meningococcal PCR results in an appropriate timely fashion.

<u>**Urgent PCR specimens**</u>

These should be discussed with a member of the MRU staff, who will liaise with colleagues in the molecular diagnostics section and make arrangements for the earliest possible testing. Contact details will be required, especially:

1. any out-of-hours contact at the sending laboratory
2. relevant CCDC or HPT.

Please <u>**do not**</u> send urgent samples likely to arrive out of hours without first discussing with MRU staff. Refer to “How to obtain our services” - ‘out of hours’ section (above).

Controlled Document

Antigen detection - non-culture confirmation

Polysaccharide antigen detection

Meningococcal antigen detection using commercial latex agglutination kits is available on request. Please discuss with a member of MRU staff before the sample is submitted.

For acute investigations, PCR is preferred as it is more sensitive and if positive, additional molecular typing can be performed.

NB: Antigen detection will reduce material available for PCR and could compromise the integrity of the sample.

What specimens to send for polysaccharide antigen detection:

CSF and serum – a minimum sample volume of 200 µL is needed.

Turnaround time

Reports will be telephoned on the day of receipt.

Printed reports will normally be sent out on the following working day.

Urgent specimen

These can be processed and results telephoned within two hours of receipt at the MRU. Please discuss with the MRU if urgent antigen tests are required.

Controlled Document

Meningococcal Serology

(a) Serodiagnosis

Serodiagnosis of meningococcal disease is not available.

(b) Pre- and post vaccine response

The following services are available via the MRU by the PHE Vaccine Evaluation Unit (VEU) based at the MMMP, Manchester Royal infirmary.

Functional, total immunoglobulin and isotype specific antibody levels for immunogenicity studies by internationally standardised assays are available.

Samples of clotted blood or serum should be collected three to eight weeks post-vaccination.

A minimum sample volume of 500µL is preferred.

There is a specific **VEU (Vaccine Preventable Serology request form)** available via the MMMP, CMFT website).
www.cmft.nhs.uk/mmmp/VEUrequestform

There will be a charge for these investigations unless they are part of an MRU or PHE instigated epidemiological investigation.

Assays available:

1. **Functional antibody to serogroups A, C, Y or W meningococci** by internationally standardised serum bactericidal assay (**SBA**).

   Where a patient's serum is tested against an international standard organism (specific to each serogroup) utilising rabbit complement in the absence of the patient's own complement.

2. **Functional antibody to serogroup B by SBA**.

   Where a patient's serum is tested against a small panel of standard meningococci (exhibiting surface vaccine antigens) utilising human complement in the absence of the patient's own complement.

Controlled Document

It is important that the patient's relevant immunisation details and any current antibiotics are written on the request form

For details of the currently available serological assays please contact VEU 0161-276-6793

The quantitation of total **IgG to serogroups C, Y, W or A polysaccharides is no longer available** for clinical samples (routine requests). It was used to support the serogroup C, Y, W or A SBA assay testing and had proved useful for earlier studies.

Charges

Requests for vaccine response testing if not initiated as part of an MRU or PHE epidemiological or case investigation: will be charged for.
Contact Mr B Wood for current prices (see below).

Turnaround Times

Functional antibody to serogroup C, Y, W, or B meningococci (vaccine response) results are available within 28 working days of submission, (see below)

.

Controlled Document

Key factors affecting specimen performance

What specimens to send

__All__ submitted PCR and Isolate **samples must comply with the sample acceptance policy** and be accompanied by a **completed MRU request form** which can be downloaded from the PHE website (or links via the MMMP, CMFT website).

__Isolates for case confirmation, epidemiology and cluster management__:

1. Please submit **__all__** available sterile site (CSF, blood and joint fluids) isolates from cases.
2. If available, please submit throat and nose swab isolates from cases as well. This is important for probable cases where blood and CSF are negative by both PCR and culture.
3. Any isolates from case contacts (nose or throat swabs) should also be sent indicating which case they relate to.

   A complete case sample set could include; CSF, blood, joint fluid, nose and throat isolates. *They are important for surveillance, molecular studies and validation of typing techniques.*

__Other non-sterile sites__:

1. Invasive respiratory samples (eg BALs), samples obtained by surgical procedure.
2. Respiratory/sputum sample isolates if thought to be clinically significant
3. *N. meningitidis* isolates with high MICs or unusual antibiograms

__GenitoUrinary Medicine (GUM) isolates__

1. Please **do not submit** routine GUM isolates

   Only submit isolates from GUM patients if they appear resistant (MICs of ≥ 0.25mg/L) or are epidemiologically linked to cases of invasive meningococcal infection.

__Other Neisseria species__

The MRU is established to confirm *N. meningitidis* and determine epidemiological markers.

1. The identification of Neisseria species other than *N. meningitidis*, *N. lactamica* and *N. gonorrhoeae* is problematic.

   Please do not submit isolates or organisms that are very unlikely to be *N. meningitidis* or *N. lactamica*. *N. gonorrhoeae*, other *Neisseria spp* and *Moraxella spp* should be referred to CfI.

Controlled Document

Turnaround Times and Price List

Meningococcal Reference Unit

	**Turn round time for Provisional result (working days)**	**Turn round time to Final result – Printed Report (working days)**
Meningococcal cultures	Provisional results are telephoned within 2 4- 72 hours	7 – 10 days
Meningococcal PCR	[1]24 hours	[2]48 hours
Meningococcal serology - serum bactericidal assay - per target	28 days	28 days

[1]PCR group B positive completed but also provisional results *ctrA* and *ply* PCR assays may available but only reported upon specific request. All positive (confirmed) results are telephoned.

[2]PCR turnaround is dependent on group confirmation algorithm and quality of the sample. All positive (confirmed) results are telephoned.

For current prices of serology (pre- and post- vaccine) assays contact Mr Bernard Wood, Head of Operations, PHE Public Health Laboratory Manchester, (bernard.wood@phe.gov.uk) for details of current prices.

Meningococcal PCR, pneumococcal PCR and isolate characterisations are not charged (for England and Wales patients) as they are regarded as of epidemiological and public health importance, particularly as part of enhanced surveillance post vaccine implantation programmes.

For meningococcal PCR prices (applicable outside England and Wales) please contact Mr Bernard Wood.

SPECIMEN ACCEPTANCE POLICY
# – GUIDANCE FOR SUBMITTING LABORATORIES AND HEALTH PROTECTION UNITS

LABELLING YOUR SPECIMENS MATTERS

Specimens **must** be correctly labelled and request forms adequately completed.
Minimise specimen rejection, confusion, delay by:

Please follow these rules:

Specimens MUST be labelled with the following:

Surname

Sender reference number

PLUS **any two out of three of the following:**
Forename
Full Date of Birth
NHS Number

AND **Date of Collection of Specimen**

Request forms MUST match the information on the sample

PLUS **Address for the report / requesting laboratory**
Patient address with postcode
Consultant, GP, CsCDC
Name of requestor
Tests required
Sender Reference Number

Request forms SHOULD have
Time and Date collected
Sex
Contact number for requestor
Relevant clinical information
Postcode

If you have any problems/queries contact: Dr Steve Gray, BMS3, MRU:
steve.gray@phe.gov.uk Tel: 0161 276 6757 / 6764 Fax: 0161 276 5744

Controlled Document

Information required

The **MRU request form MUST** be used whenever specimens are submitted**.**

This can be downloaded from the PHE website at the following link**:**

https://www.gov.uk/government/publications/meningococcal-reference-unit-request-form

Completion of the **MRU request form** ensures that the relevant investigations are carried out and reported back to sending laboratories with minimum delay. If important information is missing, sending laboratories may be contacted to supply details before testing is performed. **Please see the MRU specimen acceptance policy (above).**

It would be helpful if all requesting laboratories supplied their **telephone** and **secure fax** numbers.

The following information is important for accurate patient data reconciliation and assists provision of meaningful local statistics: **date of birth; home post code; health district of residence**.

Isolates

Please send cultures from **all** positive sites.

For all isolates:

- Presenting clinical features i.e. meningitis, septicaemia, both (if other, please give details).

Where relevant:

- Names of other possibly related cases.
- In contact tracing, the name of the index case and location (school/town etc).
- Recent travel details if there is a possibility of the disease being contracted abroad.

Meningococcal PCR

- Type of specimen – EDTA / Heparin / Serum/ CSF
- Time elapsed since illness onset
- Whether and when parenteral antibiotics have been given in relation to sample collection

Additional tests

If a named doctor requests additional tests, they can be requested by telephone or letter on samples received by the laboratory within 2 months of receipt of the sample, although it must be recognised that the archive sample available may have a limited residual volume.

Controlled Document

Complaints

Should it be necessary to submit a formal complaint to the MRU about our service please contact Dr Ed Kaczmarski or Dr Steve Gray, (see **MRU Contact Details** above).

Faxing and emailing reports containing patients' data

The following guidelines are prepared having taken into account the Code of Practice on reporting patients' results by prepared by the DoH and Caldicott recommendations.

It is MMMP (MRU) policy that reports containing patients' data, will <u>not</u> be sent by fax.

E-mails cannot be relied on to guarantee security of patients' data because they can be intercepted by a third party on route, unless encryption is used.

Therefore only secure/closed email systems may be used (phe.gov to phe.gov or nhs.net to nhs.net accounts).

Controlled Document

Compliance with the Human Tissue Act – submitting samples from deceased people

The MMMP / MRU adhere to the HTA and its application within the Central Manchester Foundation Trust site.

Tissue samples (CSF, whole blood EDTA, blood, etc.) from patients are submitted to the MRU with their consent (obtained at time of sampling) for disease confirmation, epidemiological or public health investigations. Samples are tested and retained in accordance with the MRU specimen retention policy. Original samples (following nucleic acid extraction) are kept frozen for up to one year after receipt should sufficient volume remain following initial processing.

Since 2006 in accordance with the HTA (detailed locally within MMMP-QU-PROC7), post mortem samples or samples from the deceased (patients known by the MRU to have died at the time of submission) have been returned if requested or destroyed sensitively.

A number of selected positive samples are retained for quality control, assay developments or epidemiological investigation under the local HTA guidance.

Should it be necessary to contact the MRU regarding a HTA issue, the nominated persons within MMMP are: R & D Person Designated (PD) is Professor Ray Borrow and Post Mortem PD (CMFT Virology), Dr Andrew Turner [Tel. 0161-276-5688].

MRU Recognition of Caldicott Recommendations

The recommendations of the Caldicott Report (1997) have been adopted by Public Health England and by the National Health Service as a whole. These recommendations relate to the security of patient identifying data (PID) and the uses to which they are put. MRU as an integral part of Manchester Medical Microbiology Partnership observes Caldicott guidance in handling PID. The MMMP has appointed its own Caldicott Guardian who advises on confidentiality issues and is responsible for monitoring the physical security of PID. This also applies to the transfer of results of investigations to and from MMMP whether by mail services, telephone or fax. The value of 'safe haven' arrangements or other means of the sender and receiver of information identifying themselves to each other before data are transferred is emphasized.

MMMP is anxious to audit the security of its PID in collaboration with its customers. Customers are invited to review our arrangements in conjunction with the Caldicott Guardian. Customers are also asked to draw to the Caldicott Guardian's attention any instances where PID security has been threatened or has broken down. Uses that PID are put to outside clinical diagnostic services generally allow patient identifiers to have been removed before hand, and when PID is used for research purposes the proposals are considered first by the Local (CMFT) Research Ethics Committee. All enquiries about the security and use of PID should be addressed to the MMMP Caldicott Guardian, Dr Mubby Husain (Tel: 0161-701-4774); e-mail mubby.husain@phe.gov.uk.

Controlled Document

Key References

- Borrow R, Carlone GM. 2001. Seogroup B and C serum bactericidal assays. In: *Meningococcal Vaccines. Methods in Molecular Medicine* (eds. Pollard AJ and Maiden MCJ) pp 289 – 308. Humana Press, Totowa, New Jersey.
- Cartwright K. Meningococcal Disease. J Wiley & Sons 1995.
- Corless CE, Guiver M, Borrow R, Edwards-Jones V, Fox AJ, Kaczmarski EB. 2001. Simultaneous detection of Neisseria meningitidis, Haemophilus influenzae, and Streptococcus pneumoniae in suspected cases of meningitis and septicemia using real-time PCR. *J Clin Microbiol*. **39**:1553-8.
- Dagan R, Shriker O, Hazan I, Leibovitz, Greenberg D, Schlaeffer and Levy R. 1998. Prospective Study to Determine Clinical Relevance of Detection of Pneumococcal DNA in Sera of Children. J*. Clin. Microbiol*. March 1998 36: 3; 669-673.
- Feavers IM, Fox AJ, Gray SJ, Jones DM and Maiden MC. 1996. Antigenic diversity of meningococcal outer membrane protein porA has implications for epidemiological analysis and vaccine design. *Clin. Diagn. Lab. Immunol* **3**(4): 444-450.
- Gray SJ, Borrow R, Kaczmarski EB. 2001. Meningococcal serology. In: *Meningococcal Disease. Methods in Molecular Medicine* (eds. Pollard AJ and Maiden MCJ) pp 61 – 87. Humana Press, Totowa New Jersey.
- Gray SJ, Trotter CL, Ramsay ME, Guiver M, Fox AJ, Borrow R, Mallard RH and Kaczmarski EB. 2006. Epidemiology of meningococcal disease in England and Wales 1993/94 to 2003/04: contribution and experiences of the Meningococcal Reference Unit. J Med Microbiol **55**: 887-896
- Gray SJ, Guiver M, Lord A, Borrow R, Kaczmarski EB and McHugh M EB. 2012. Quality improvements for the non-culture (PCR) confirmation of meningococcal disease in England. XVIIIth International Pathogenic Neisseria Conference (IPNC), Wuerzburg, Gemany, 9–14th September 2012. http://neisseria.org/ipnc/2012/IPNC_2012_abstracts.pdf. Poster abstract 121, page 261
- Guiver M, Borrow R, Marsh J, Gray SJ, Kaczmarski EB, Howells D, Boseley P, Fox AJ. 2000. Evaluation of the Applied Biosystems automated Taqman polymerase chain reaction system for the detection of meningococcal DNA. *FEMS Immunol Med Microbiol*; **28**: 173-179.
- Guiver M and Borrow R. 2001. PCR diagnostics. In: *Meningococcal Disease. Methods in Molecular Medicine* (eds. Pollard AJ and Maiden MCJ) pp 23 – 39. Humana Press, Totowa, New Jersey.
- Guiver M, Corless CE, WJ Marsh, Gray SJ, Newbold LS, Borrow R and Kaczmarski EB. 2011. Modifications to a Published *ctrA* PCR Assay for the Improved Non-Culture Confirmation of Meningococcal Disease in England and Wales. Meningitis Research Foundation meningitis and septicaemia in children and adults conference 4-5th November 2011, London, UK. Poster abstract. http://www.meningitis.org/posters
- Hackett SJ, Guiver M, Marsh J, Sills SA, Thomson APJ, Kaczmarski EB and Hart CA. 2002. Meningococcal bacterial DNA load at presentation correlates with disease

Controlled Document

severity. *Arch Dis Child* 2002;86:44-46 doi:10.1136/adc.86.1.44

- Jolley K, Urwin R, Suker J, and Gray SJ. 2006. Methods for meningococcal typing in Handbook of Meningococcal Disease: Infection, biology and Clinical Management. Editors M Frosch and MCJ Maiden. Chpt3; 37-51. Wiley-VCH, USA.
- Kaczmarski EB. Meningococcal disease in England and Wales: 1995. *Comm Dis Rep Rev* 1997; **7**: R55-R59.
- Kaczmarski EB, Cartwright KAV. 1997. Control of meningococcal disease: guidance for microbiologists. *Comm Dis Rep Rev* **5**: R196-R198.
- Kuipers, B., van den Dopplesteen, G., Wedege, E. & van Alphen, L. 2001. Serological characterization. In *Meningococcal Disease: methods and protocols*. pp131-145. Edited by Pollard, A.J. & Maiden, M.C.J. Humana Press Inc., Totowa, New Jersey, USA.
- McHugh MP, Gray SJ, Kaczmarski EB, Guiver M. 2015. Reduced Turnaround Time and Improved Diagnosis of Invasive Serogroup B *Neisseria meningitidis* and *Streptococcus pneumoniae* Infections Using a Lyophilised Quadruplex qPCR. JMM [*Epub ahead of print 06 August 2015* 10.1099/jmm.0.000154
-
- Ragunathan L., Ramsay M, Borrow R, Guiver, M, Gray S and Kaczmarski EB. 2000. Clinical features, laboratory findings, and management of meningococcal meningitis in England and Wales: report of a 1997 survey. Meningococcal meningitis: 1997 survey report. J. Infect. **40**:70-79.
- Rosenqvist, E., Wedege, E., Hoiby, E.A. & Froholm, L.O. 1990. Serogroup determination of *Neisseria meningitidis* by whole-cell ELISA, dot-blotting and agglutination. *APMIS* 1990; **98**: 501-506.
- Suker J, Feavers IM and Maiden MC. 1996. Monoclonal antibody recognition of members of the meningococcal P1.10 variable region family: implications for serological typing and vaccine design. *Microbiology* **142**: 63-69.
- Wedege, E., Hoiby, E.A., Rosenqvist, E. & Froholm, L.O. 1990. Serotyping and serosubtyping of *Neisseria meningitidis* isolates by co-agglutination, dot-blotting and ELISA. *J Med Microbiol* 31: 195-201.
- WHO. 2011. "Laboratory Methods for the diagnosis of meningitis caused by *Neisseria meningitidis*, *Streptococcus pneumonia*e and *Haemophilus influenza*e. WHO Manual, 2nd Edition. Ed. L Meyer. WHO/IVB.11.09. p266-268.

Controlled Document

Charities and Public Information Contact Details

Meningitis Research Foundation (MRF)
Midland Way
Thornbury
Bristol
BS35 2BS

Tel: +44 (0) 01454 281811
Fax: +44 (0) 01454 281094

Email: info@meningitis.org

http://www.meningitis.org/

Meningitis Now - Head Office,
Fern House,
Bath Road,
Stroud,
Gloucestershire
GL5 3TJ,
United Kingdom

Tel: +44 (0)1453 768000
Fax: + 44 (0)1453 768001

Email**:** info@meningitisnow.org

Emails to this address are only answered Monday - Friday 9am - 5pm.
If your question is urgent, please call our Helpline.

Nurse-led Helplines
UK: Freephone 0808 80 10 388
International: +44 (0)1453 768002

https://www.meningitisnow.org/

Controlled Document
